# The Importance of *STK11*/*LKB1* Assessment in Non-Small Cell Lung Carcinomas

**DOI:** 10.3390/diagnostics11020196

**Published:** 2021-01-29

**Authors:** Baharia Mograbi, Simon Heeke, Paul Hofman

**Affiliations:** 1 Centre Antoine Lacassagne, CNRS, FHU OncoAge, Team 4, INSERM, IRCAN, Université Côte d’Azur, 06000 Nice, France; mograbi@unice.fr; 2 Department of Thoracic Head and Neck Medical Oncology, UT MD Anderson Cancer Center, Houston, TX 77030, USA; shj.heeke@gmail.com; 3 CHU Nice, Laboratory of Clinical and Experimental Pathology, FHU OncoAge, Pasteur Hospital, Université Côte d’Azur, 06000 Nice, France; 4 CHU Nice, FHU OncoAge, Hospital-Integrated Biobank BB-0033-00025, Université Côte d’Azur, 06000 Nice, France

**Keywords:** non-small cell lung carcinoma, immunotherapy, targeted therapy, *STK11/LKB1*, *KRAS*, biomarker

## Abstract

Despite the recent implementation of immunotherapy as a single treatment or in combination with chemotherapy for first-line treatment of advanced non-small cell lung cancer (NSCLC), many patients do not benefit from this regimen due to primary treatment resistance or toxicity. Consequently, there is an urgent need to develop efficient biomarkers that can select patients who will benefit from immunotherapy thereby providing the appropriate treatment and avoiding toxicity. One of the biomarkers recently described for the stratification of NSCLC patients undergoing immunotherapy are mutations in *STK11/LKB1*, which are often associated with a lack of response to immunotherapy in some patients. Therefore, the purpose of this review is to describe the different cellular mechanisms associated with *STK11/LKB1* mutations, which may explain the lack of response to immunotherapy. Moreover the review addresses the co-occurrence of additional mutations that may influence the response to immunotherapy and the current clinical studies that have further explored *STK11/LKB1* as a predictive biomarker. Additionally this work includes the opportunities and limitations to look for the *STK11/LKB1* status in the therapeutic strategy for NSCLC patients.

## 1. Introduction

Over the past few years, the mortality due to non-small cell lung cancer (NSCLC) decreased significantly, mainly due to early diagnosis, but also to the development of new therapeutic strategies, including targeted therapies [1]. Additionally, the introduction of immunotherapy alone or in combination with chemotherapy has had a dramatic impact on the prognosis of patients by providing a substantial improvement in overall survival [2]. In this context, many clinical trials have evaluated immunotherapy in the first- and second-line settings with the aim to develop a more efficient treatment strategy with less toxicity [3,4,5,6,7,8,9,10,11].

In line with the tremendous clinical progress, the understanding of the efficacy of immunotherapy in NSCLC and its pathophysiology has unraveled new cellular mechanisms associated with its response to treatment and to intrinsic resistance [12,13,14,15]. Moreover, bioinformatic analyses are becoming increasingly sophisticated allowing the analysis and integration of complex clinical and biological data to further understand the biology of cancer, notably of lung carcinoma [16,17,18,19]. Unfortunately, and despite recent progress, the development and clinical validation of novel robust biomarkers that predict response, resistance and/or toxicity to the treatment in routine clinical care remain major challenges in thoracic oncology.

Many research studies and clinical trials have evaluated the potential of different prognostic and predictive biomarkers in thoracic oncology [20,21,22,23,24,25,26,27,28,29,30,31]. Importantly, an increasing number of biomarkers have been developed and evaluated specifically to determine the response to immunotherapy in NSCLC, including a few developed specifically to assess toxicity [27,31,32,33,34,35,36,37,38,39,40,41,42,43,44,45,46,47,48,49]. These biomarkers demonstrated caveats that limit their implementation [27,34,36,38,40].

Recently, different studies have associated the presence of *STK11* mutations with a lack of response to immunotherapy in NSCLC [50,51,52,53,54]. Additionally, some preclinical and translational studies have shed further light on the biological role of *STK11* mutations leading to primary resistance to immunotherapy [55,56,57]. Nevertheless, the implementation of *STK11* mutations as a routine biomarker in NSCLC, in addition to the current mandatory or recommended therapeutic targets (*EGFR*, *ALK*, *ROS1*, *BRAF*, *NTRK*, PDL-1, *RET*, *MET* and *HER2*), remains controversial and is not performed in daily practice [58].

Therefore, this review aims to highlight the current research of *STK11* mutations in late stage NSCLC, the considerations for its potential implementation in routine clinical care, and finally the current limitations of using the *STK11* mutational status in decision making of the global therapeutic strategy in thoracic oncology.

## 2. The Double-Edged Sword of *STK11* in Cancer Cell Metabolism

**Figure 1:**
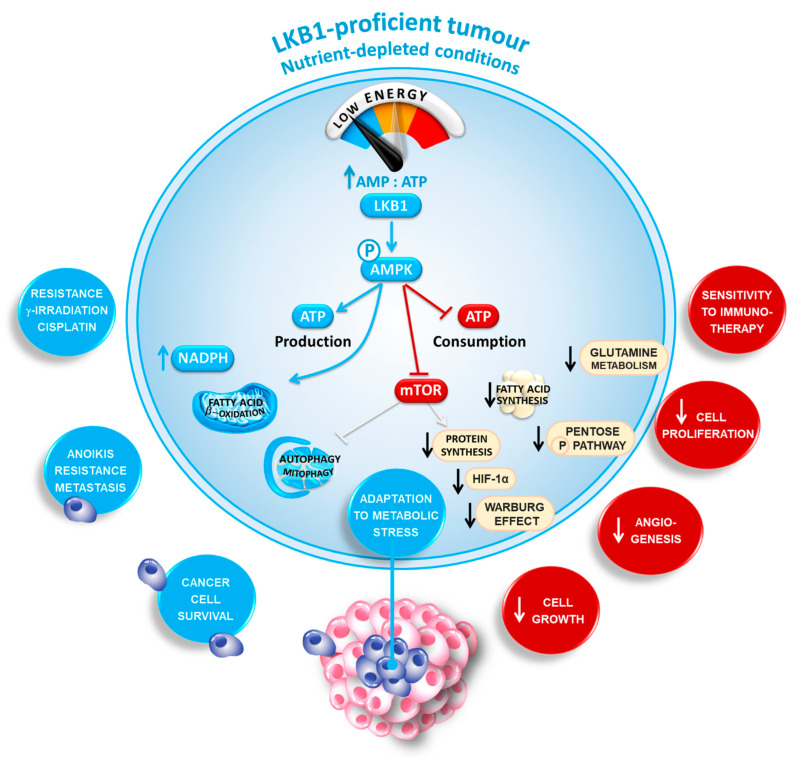
The double-edged sword of liver kinase B1 (LKB1) in cancer cell metabolism. LKB1 is a master metabolic sensor that acts as an energy gauge to sustain cancer cell survival. Upon nutrient deprivation within the center of large tumors, LKB1 reprograms cell metabolism by slowing down all ATP-consuming processes while simultaneously stimulating ATP-generating processes. By organizing this overall stress response, LKB1 may adapt cancer cell growth under conditions of energy shortage.

The *STK11* gene is located on the short arm of chromosome 19 (19p13.3) and its nine exons codes for liver kinase B1 (LKB1), a protein kinase and master metabolic sensor that acts as an energy gauge to sustain cancer cell survival [59,60,61]. Upon nutrient deprivation, as occurs often in the center of large tumors, LKB1 orchestrates cell metabolism by reducing ATP-consuming processes while simultaneously stimulating ATP-generation. Mechanistically, LKB1 is the upstream serine/threonine kinase for the AMP-activated protein kinase (AMPK). Activation of AMPK redirects metabolism towards decreased fatty acid (FA) synthesis, increased glycolysis, and FA oxidation to replenish ATP stores [60,62,63,64,65,66]. Consequently, LKB1 activates several downstream kinases of the AMPK family by direct phosphorylation in the T-loop domain [60]. In particular, the activation of AMPK upon energetic stress has been intensively analyzed in various diseases, including cancer to induce a metabolic switch from anabolism towards catabolism to regulate energy homeostasis and cell survival (Figure 1).

Besides its “classical” role as a metabolic checkpoint inhibitor, AMPK directly phosphorylates and inhibits the mammalian target of rapamycin (mTOR), shutting down protein synthesis, the cellular process that consumes the most ATP [67]. Inhibition of mTOR downregulates hypoxia-inducible factor 1α (HIF-1α), thereby negatively affecting angiogenesis and anaerobic glycolysis (the “Warburg effect”) [68]. Advances in molecular profiling have identified mutations or amplifications of specific genes involved in the mTOR pathway (e.g., *PIK3CA*, *PTEN*, *STK11* and *RICTOR*) as the most common mechanism leading to mTOR hyperactivation [69]. Noteworthy, LKB1, AMPK and mTORC1 are recruited onto the lysosomal surface upon glucose deprivation where they inhibit mTOR [70]. This activation of the LKB1/AMPK/mTOR signaling pathway on lysosomes commits the cells to basically cannibalize themselves via the autophagy-lysosomal pathway [71,72]. Indeed, suppression of the activity of mTORC1 releases the inhibitory phosphorylation on Unc-51-Like Autophagy Activating Kinase 1 (ULK1), a kinase essential for initiation of autophagy [73]. As a safeguard, AMPK also directly phosphorylates and activates ULK1 and the proautophagy lipid kinase VPS34 [74,75]. Finally, inactivation of mTORC1 releases the inhibitory phosphorylation of TFEB and TFE3 [76]. Simultaneously AMPK directly phosphorylates and activates the transcription factors FOXO and PGC-1α (peroxisome proliferator-activated receptor-gamma coactivator 1α) [77,78]. TFEB, TFE3, PGC-1α and FOXO translocate to the nucleus, where they drive the expression of genes involved in autophagy, mitochondrial processes and lysosomes. Autophagy may then help the cell degrade dysfunctional mitochondria (mitophagy) to fuel the nutrients required for oxidative metabolism [79].

It is also critical to highlight that LKB1/AMPK may safeguard against oxidative stress by inhibiting NADPH-consuming FA synthesis and increasing NADPH-producing FA oxidation [80]. Meanwhile, activated AMPK also phosphorylates and activates the transcription factor NRF2 [81]. NRF2 then activates the transcription of antioxidant genes involved in the production of NADPH. The high NADPH levels, together with autophagy, protect the LKB1-proficient cancers from oxidative stress and ROS-inducing chemotherapies (cisplatin, paclitaxel and doxorubicin) [59]. Consequently, NRF2 activation has been associated with more aggressive lung cancer and decreased survival in patients [82]. Interestingly, the activation of the LKB1/autophagy pathway enables circulating tumor cells to resist anoikis [83]. Accordingly, cells lacking LKB1 undergo apoptosis under metabolic stress as they are unable to respond to a deficiency in energy and restore homeostasis [64].

By orchestrating this overall stress response, LKB1 may help cancer cells to continuously “fine-tune” their growth rate in response to fluctuations in energy in their environment. As such, these dual pro- and antitumoral roles of LKB1 implicitly suggest that LKB1 is not always functioning as a tumor suppressor, as was initially thought and may link LKB1 signaling to tumor progression. In addition to AMPK two other AMPK-related kinases, the salt-inducible kinases SIK1 and SIK3, emerge as predominant downstream targets of LKB1 in NSCLC [84,85]. It seems of great interest to look for the impact of these different pathways on the potential efficiency of the immune checkpoint inhibitors (ICIs), notably those targeting PD-L1/PD1 [2,12,13,14]. Hence, many developments have granted a clearer perceptive on the different factors that reduce an antitumor immune response, leading to the discovery of several agents that work on immune costimulatory and inhibitory checkpoint pathways. So, the best examples of the advanced checkpoint molecules that mediate tumor-induced immune suppression are PD-1 and PD-L1 [12,13,14]. Whether the SIK-dependent pathway plays a role in the resistance to the ICIs however, remains to be determined.

**Figure 2:**
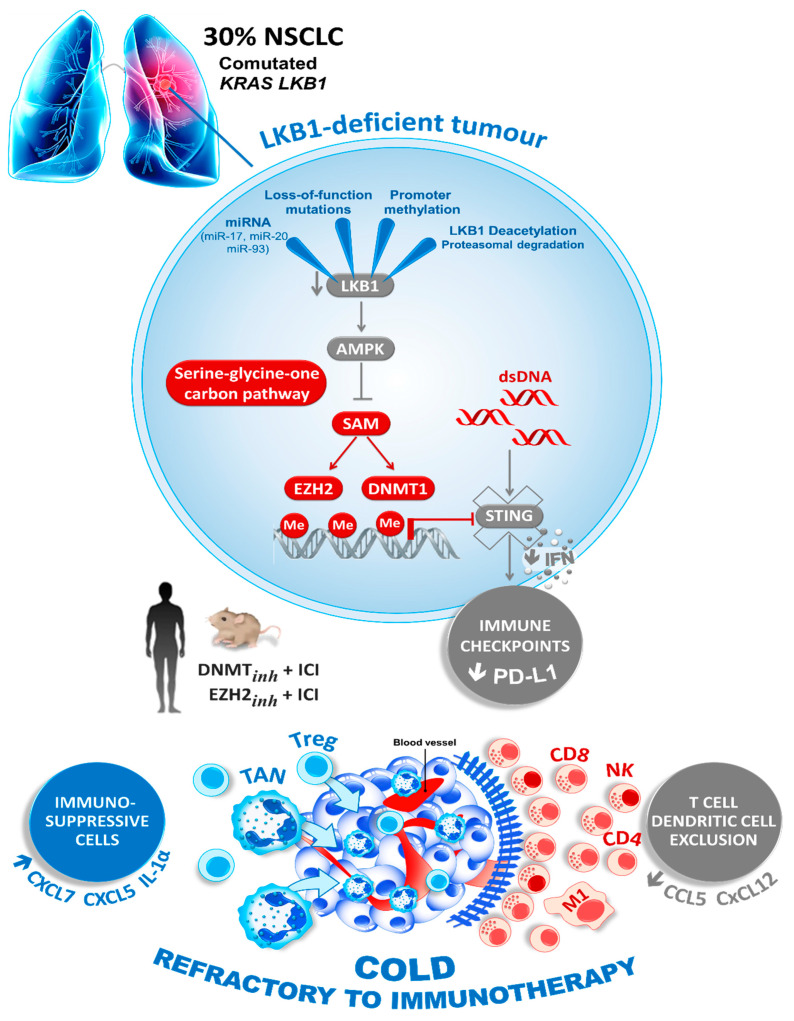
Loss of LKB1 drives the tumor immune escape. The loss of LKB1, the second most commonly altered tumor suppressor in NSCLC, promotes the production of SAM, a substrate for the DNA and histone methyltransferases DNMT1, EZH2 and other epigenetic silencing enzymes. This downregulates the expression of STING, impairing dsDNA sensing, and thereby the expression of immune checkpoint regulating proteins like PD-L1 and T cell chemokines. Therefore, the LKB1-deficient tumors are characterized by a so-called “cold” immunosuppressive tumor microenvironment (blue), which shows the striking infiltration of immunosuppressive cells (TAN; tumor-associated neutrophil; Treg, T lymphocyte regulator, blue) and the exclusion of inflamed immune cells (CD8 T cells, NK, CD4 T cells and M1; Macrophage type 1, red). Epigenetic therapies that reactivate LKB1 or the STING pathway may boost an anticancer immune response in LKB1-deficient cancers with the resistance to immune-checkpoint blockade (ICI, immune checkpoint inhibitor).

More recently, a connection between *STK11* expression and the stimulator of interferon genes (STING) pathway was also highlighted [55,86]. STING is a cytosolic protein activated by the presence of free double-stranded DNA (dsDNA) in the cytoplasm, due to pathogen infection or neoplastic transformation. Aberrant cytoplasmic dsDNA activates STING oligomerization on the endoplasmic reticulum–Golgi membrane. STING then recruits and activates TBK1 (tank-binding kinase 1), which phosphorylates the transcription factor IRF3 to induce the production of type-I interferons and other chemokines, and finally the T cell recruitment. In cancer, STING may play a crucial role as one of the initial steps needed for immune evasion. Interestingly, Kitajima et al. described the downregulation of STING in *KRAS*/*STK11* comutated cancer cells [87]. *STK11* inactivation dysregulates serine metabolism, leading to increased levels of S-adenosyl methionine (SAM). SAM is a substrate for multiple epigenetic silencing enzymes, such as DNMT1 and EZH2, that are both directly involved in the methylation of the STING promoter, causing its down-modulation and repression [87] (Figure 2).

Furthermore, the regulation of the expression of *STK11* and its role in cancer cell proliferation remain very complex [88]. Recent studies for example showed that asparagine and aspartate could regulate AMPK-mediated p53 activation by physically binding to LKB1 and modulating LKB1 activity. It seems that p53 can regulate asparagine metabolism to control cell survival by generating an auto-amplification loop via asparagine-aspartate-mediated LKB1-AMPK signaling [88].

## 3. *STK11* and Associated Genomic Alterations in Lung Cancer

*STK11* mutations are present in many different tumor types but with varying frequencies [89]. They are more frequently observed in NSCLC, notably in lung adenocarcinoma [89,90,91]. However, reports of the frequency of mutations in lung cancer vary in the literature, and it is also different depending on the patient ethnicity [91,92,93,94,95]. *STK11* mutations are less frequent in Asian (4–7%) than in Caucasian patients (16%) in NSCLC analyzed by the TCGA project [96]. They were also significantly higher among Afro-American patients [97]. Interestingly, in one series only 77/1385 (6%) NSCLC patients harbored an *STK11* mutation according to next generation sequencing (NGS) analysis while the AACR genie database (v8.1 public) reported *STK11* mutations in 1495/14,303 (10.5%) of samples [92]. In addition to lung adenocarcinoma, some pulmonary large-cell neuroendocrine carcinomas can also harbor *STK11* mutations [98,99,100]. The most frequently comutated genes are highlighted in Table 1.

Importantly, these *STK11* mutations have to be analyzed in association with other comutations of interest, notably in *KRAS*, *KEAP1*, *TP53* and *SMARCA4* [92,101,102]. Arbour et al. showed that among 330 patients with late stage *KRAS*-mutant NSCLC, the most frequent comutations were found in *TP53* (42%), *STK11* (29%) and *KEAP1*/*NFE2L2* (27%) [101]. Furthermore, in 62 patients with *STK11* mutated tumors analyzed by Bange et al., 18 had an *STK11* mutation alone, while 19, 18 and 7 patients had *STK11*/*KRAS*, *STK11*/*TP53* and *STK11*/*KRAS*/*TP53* comutations, respectively [92]. In contrast, a recent study analyzing 69 patients showed that the mutations in *STK11* were more frequently observed in the *KRAS* wild type population than in the *KRAS* mutated tumors [103]. Additionally, *SMARCA4* mutations were associated with *STK11* mutations in 39% of all cases [104]. *SMARCA4* mutations can be classified into class-I with *SMARCA4* truncating mutations, some fusions and homozygous deletions, and into class 2 with *SMARCA4* missense mutations or variants of unknown significance [104]. Importantly, *STK11* mutations are mainly associated with *SMARCA4* class-I mutations [104]. Moreover, the loss of *BRG1* expression, which can be detected by immunohistochemistry with an anti-BRG1 antibody, is associated with *SMARCA4* class I mutations, and was found to be predominantly detected in adenocarcinomas with co-occurring mutations in *KRAS*, *STK11*, *TP53* and *KEAP1* [105]. Other mutations or gene activations, such as *NFE2L2* mutations are also often associated with *STK11* mutations [82,106]. It was recently demonstrated that NRF2 activation acts as a critical oncogenic driver, and can promote aggressive lung adenocarcinoma by cooperating with *STK11* loss and *KRAS* activation [82]. Additionally, patients with NRF2-activated non-squamous or squamous tumors have a poor prognosis and limited response to anti-PD-L1 treatment [82,107].

## 4. *STK11* as a Prognostic Biomarker in Lung Cancer

The presence of *STK11* mutations in association with additional mutations in *KRAS*, *KEAP1* and *SMARCA4* has been reported as an independent negative prognostic factor for overall survival in NSCLC patients [102]. Different studies reported clusters of *KRAS*-mutant lung adenocarcinomas bearing *STK11* mutations, *TP53* mutations or *CDKN2A*/*B* inactivation [108,109]. In additional studies, patients with a *KRAS*/*STK11* comutation had a poorer overall survival than *KRAS* mutated patients without a *STK11* mutation or with *KRAS*/*TP53* comutations [50,92,110]. La Fleur et al. showed that patients with an isolated *KRAS* mutation had an overall survival similar to those of the wild-type group, whereas patients with co-occurring mutations in either *TP53* or *STK11* had a worse overall survival in comparison to the wild-type group and the *KRAS* mutated only group [110]. Conversely other studies showed that the tumors with comutated *KRAS* and *STK11* did not have a worse prognosis than *KRAS* mutated/*STK11* wild-type tumors [101]. Moreover, the analysis of patients’ survival with early stage lung adenocarcinoma in the TCGA PanCancer data set also highlighted poorer overall survival in patients with *STK11* mutations. Additionally, a recent study showed that patients with *STK11* mut/*KRAS* wt tumors had a worse prognosis compared to patients with *STK11* and *KRAS* double mutated tumors [103].

## 5. *STK11* as a Predictive Biomarker for the Therapy Response in Lung Cancer

While many studies have highlighted the prognostic value of *STK11* mutations, it remains unclear if *STK11* mutations are also predictive of response to immunotherapy. In some series of NSCLC with *STK11* mutations treated with first-line systemic therapy, a comutation with *KRAS* was associated with significantly worse progression-free survival and overall survival [92]. In contrast, comutation of *STK11* with *TP53* conferred a better prognosis in these patients [92]. 

Additionally, another study showed a higher prolonged progression-free survival in anti-PD-1-treated patients harboring *TP53*-mut/*STK11*-*EGFR*-wt tumors than in patients with *TP53*-wt/*STK11*-mut [111]. A few initial studies demonstrated that mutated *STK11* tumors, notably with *TP53*, *KRAS* and *KEAP1* mutations, showed primary resistance to ICIs [15,53,57]. Using whole-exome sequencing to examine NSCLC patients treated with PD-1 plus CTLA-4 blockade, a study demonstrated that a couple of patients with a *STK11* mutation had primary resistance to this therapeutic combination [6]. Most of the studies demonstrated a low level of expression of PD-L1 in patients with a *STK11* mutation, which could explain the resistance to immunotherapy [112,113]. In the study of Lamberti et al., the PD-L1-negative group, compared with the PD-L1-high group, had a higher number of mutations in *STK11* (19% versus 6%) [113]. In contrast, other studies reported that lung cancer patients with *STK11* mutated tumors could respond to ICIs [114,115,116]. However, the response could sometimes be associated with co-occurring *TP53* mutations [86,116]. Additionally, a retrospective analysis of the Keynote-042 trial, which evaluated pembrolizumab vs. platinum-based chemotherapy in NSCLC in the first-line setting, revealed comparable response rates between *STK11* mutated and *STK11* wild-type tumors [5]. In line, an analysis of 2276 NSCLC patients treated in first-line with ICIs demonstrated that the presence of comutations in *STK11* and *KEAP1* were prognostic rather than predictive [117]. Additionally, a recent study showed that despite the presence of a *STK11* mutation, the presence of *SMARCA4* mutations could have a positive predictive value for ICI responsiveness, highlighting the controversial role of *STK11* as a predictive biomarker [102].

Interestingly, a genomic mutation signature (GMS) obtained with eight selected genes (*TP53*, *KRAS*, *STK11*, *EGFR*, *PTPRD*, *KMT2C*, *SMAD4* and *HGF*) was able to predict response of NSCLC patients to immunotherapy, most notably to anti-PD-1 therapy [118]. The GMS was independent of TMB and PD-L1 expression and predicted response across different clinico-pathological features and combining PD-L1 expression with the GMS improved prediction of response [118].

In addition to the evaluation of *STK11* mutations, another study examined the expression of LKB1 using immunohistochemistry. LKB1 expression in more than 50% of tumor cells was defined as LKB1 high and results correlated to the efficacy of pembrolizumab monotherapy in untreated patients with advanced NSCLC [119]. In this study, the median progression free survival and overall survival of patients was numerically shorter in the cohort with low LKB1 expression, although the results were not statistically significant [119]. However, only a few patients were included in this study. Most importantly, this study did not assess *STK11* mutations and thus the relationship between expression and mutational status of *STK11* remains unknown [119]. It would have been of interest to see how the expression of *STK11* differed in the context of the presence of other comutations like *KEAP1* or *KRAS* and to analyze the tumor microenvironment, as the composition of different immune cells might be different among different *KRAS* mutations [120].

Despite the association of *STK11* mutations with response to immunotherapy, some studies have also evaluated *STK11* mutations in populations that were treated with chemotherapy. In a French cohort of patients receiving chemotherapy in first-line, 25/302 (8%) NSCLC had a *STK11* mutation [93]. No statistical difference was observed between the *STK11* status and clinico-pathological variables. Overall survival was shorter for *STK11* mutated patients in a univariate analysis. However, the *STK11* status did not have an impact on overall survival in a multivariate analysis and progression free survival was not significantly different between the populations [93]. Interestingly one patient with both *STK11* and *NFE2L2* mutations had a good response to platinum-based chemotherapy [106]. In contrast, a recent study from Jeong et al. demonstrated that deletion of *KEAP1* conferred chemoresistance in preclinical models of lung adenocarcinoma and that patients with late stage NSCLC with *KEAP1*/*NFE2L2*/*CUL3* mutations had a shorter time to treatment failure and overall survival when treated with front-line platinum doublet chemotherapy [121]. However, the impact of *KRAS* and *STK11* mutations on these results were not estimated [121].

## 6. Potential Treatments Targeting *STK11* Mutations in Lung Cancer

To overcome *STK11* mediated resistance to immunotherapy, novel compounds are being developed to target this distinct group of lung cancers [2]. Among them, the recently emerging *KRAS* inhibitors that currently target preferentially *KRAS* G12C are actively being investigated [122,123,124]. Importantly, considering the impact of different comutations together with *KRAS*, an important source of heterogeneity in *KRAS* mutated lung cancer, current drug development programs need to take this complexity into account and design studies that also consider the comutations [125]. However, the impact of a *KRAS* inhibition on a *STK11* comutated tumor remains unknown and different alternative approaches might be necessary to better target these tumors [89,126]. For example, metformin, phenformin with or without sapanisertib, everolimus, tunicamycin, brefeldin A or 2-deoxyglucose, which target different metabolic pathways that can be altered by *STK11* mutations have been actively studied [89]. Additionally, a recent study demonstrated that ERK inhibitors can be effective in *STK11* mutated tumors in vitro, and which was proposed as a novel therapeutic strategy [127]. Interestingly, *STK11* loss induced an increase in energetic/redox stress, which is tolerated, in part, through co-occurring KEAP1/NRF2-dependent metabolic adaptations, thus enhancing glutamine dependence, which rendered those tumors sensitive to glutaminase inhibitors [128]. This was further supported by new research using a combination of CRISPR-Cas9-based genetic screening and metabolomic analyses, which further confirmed that *KEAP1* or *NRF2*-mutant cancers are dependent on increased glutaminolysis, which can be therapeutically exploited through the pharmacological inhibition of glutaminase [129].

## 7. Assessment of the *STK11* Status in Lung Cancer

Currently the detection of *STK11* mutations is mainly performed using NGS with gene panels of different sizes (from a few genes to large panels of 500 genes) and both tissue and liquid biopsies have been used as testing material [116,130,131]. In the context of *STK11* mutations, it is particularly important to carefully consider the most effective panel size for an NGS panel due to the particular importance of comutations in this context [105]. Some genes of interest, such as *KEAP1* and/or *SMARCA4* but also other genes that are often comutated with *STK11* are absent in some small panels [132,133,134,135,136]. Additionally, *STK11* itself may be absent with most of the rapid sequencing approaches, highlighting the importance of the careful selection of the appropriate gene panels [135].

Therefore, it might be more suitable to integrate larger gene panels for NGS testing when the *STK11* status is of interest to carefully assess all important comutations and not just the currently mandatory genes for baseline assessment in NSCLC.

## 8. Integrating the *STK11* Mutation Status in the Treatment of Lung Cancer

Identifying clinical or molecular factors that predict the benefit of checkpoint inhibitors in advanced NSCLC remains crucial for the selection of appropriate therapies for each patient. Currently, the expression of PD-L1 on tumor cells remains the principal biomarker to predict the efficacy of checkpoint inhibitors on NSCLC and other tumors. Although there is a linear relationship between the level of the benefit of checkpoint inhibitors and the level of tumor PD-L1 expression in NSCLC, a response of tumors to immunotherapy has still been observed in those with low or undetectable PD-L1 expression [36,116,137]. In contrast, tumors may be unresponsive to immunotherapy even when expressing high levels of PD-L1, highlighting the limitations of PD-L1 for therapy selection [36,138]. While the value of the tumor mutational burden (TMB) has been extensively studied for the prediction of response to immunotherapy, which led recently to its highly debated approval by the FDA, TMB is still a controversial biomarker [21,34].

So far, the assessment of *KRAS* mutations has been considered to be of interest before chemotherapy and/or ICI treatment, but recent studies demonstrated that the assessment of *KRAS* alone might be of limited value [139]. Moreover, many studies have found a strong association between mutations in *KRAS* and *STK11* [140].

Considering the challenges of using biomarkers for the selection of immunotherapy for patients with NSCLC, it may be of interest to integrate the *STK11* status for late stage NSCLC in patients eligible for treatment with first-line ICIs alone or in association with chemotherapy. In this regard, it was proposed that other genes, such as *KEAP1* should now be evaluated and also associated [141]. Consequently, one could theoretically argue that patients with tumors showing an expression of PD-L1 in more than 50% of tumor cells, without other druggable mutations in *EGFR*, *ALK*, *ROS1*, *BRAF*, *NTRK*, *RET*, *HER2* and *MET*, but having an *STK11* mutation, should not be treated with ICIs, and should potentially only receive chemotherapy [57]. However, it is very important in this context to highlight the limitations of using *STK11* mutations for patient stratification as discussed above. Most importantly, it remains unclear if *STK11* can serve as a predictive biomarker that can guide treatment selection and prospective evaluation is still missing. Consequently, immunotherapy should not currently be withheld from patients with *STK11* mutated tumors. However, in the case of rapid tumor progression under treatment or in the case of a non-tumoral response, consideration of a *STK11* mutation may allow rapid adaptation of therapy for these patients [105]. Additionally, *STK11* expression could also be considered [142,143]. However, multiplexing different antibodies of interest for diagnosis and/or assessment of predictive markers may be needed to reserve material for further genomic studies [144,145]. For first-line immunotherapy in NSCLC, future developments should at the same time integrate not only several genomic alterations of interest, including *STK11* mutation, but also the assessment of the expression of different proteins and cytokines in the same sample, knowing the impact of different associated mutations in the tumor environment [56,116,120].

## 9. Conclusions

The in vitro research and clinical trials that integrate evaluation of genomic alterations for treatment responsiveness and comparison of efficacy of different molecular strategies have opened up new approaches to lung cancer precision medicine. However, there is an urgent need to integrate simultaneously many biomarkers to propose the best therapeutic strategy for lung cancer patients [58]. These different biomarkers constitute an increasing number of genomic alterations, identified not only in tissue and cytological samples, but also in liquid biopsies [116,146,147,148]. Recent research has expanded dramatically our understanding of the role of *STK11* mutations in mediating resistance to anticancer immunotherapy in NSCLC and have revealed novel therapeutic approaches both in vitro and in vivo. However, these recent studies also highlighted the complexity of the biology of NSCLC and the importance of also assessing co-occurring mutations, which influence the response to therapy. Therefore, it seems essential to evaluate the status of combinations of different genes such as *STK11*, *TP53*, *KRAS*, *KEAP1* and *SMARCA4*, to cite a few, and the different classes of genomic alterations present on these different genes. These analyses will certainly extend our understanding of resistance to immunotherapy and will improve the selection of appropriate therapies for personalized medicine of patients with NSCLC.

## Figures and Tables

**Table 1:** Most frequently comutated genes with *STK11* in non-small cell lung cancer according to AACR Genie Database (v8.1-public)**.**

Non-small cell lung cancer ^§^ (*n* = 14.300)	**Gene**	**Samples Mutated/Samples Tested** **^ǂ^**	**Percentage of Samples Comutated with *STK11***
	*STK*	1495/1535 ^¥^	97.4%
	*KRAS*	760/1535	49.5%
	*KEAP1*	618/1255	49.2%
	*TP53*	626/1535	40.8%
	*SMARCA4*	261/1329	19.6%
	*ATM*	209/1534	13.6%

^§^ The majority of the samples (11.107/14.300) were adenocarcinoma. ^ǂ^ Samples tested for the comutations differs in the AACR Genie database as not all genes are present on the different method tested. ^¥^ 1535 samples of 1495 patients were tested. All patients had at least one sample with a *STK11* mutation and thus all patients were defined to be *STK11* positive.

## Data Availability

Not applicable.
